# Alzheimer’s disease susceptibility in African American elders: a classification and regression tree (CART) analysis approach

**DOI:** 10.58398/0001.000008

**Published:** 2023-12-31

**Authors:** Sung Seek Moon, Lindsey Anderson, Jinwon Lee, Youngkwang Moon

**Affiliations:** a Diana R. Garland School of Social Work, Baylor University, Texas, USA; b Creative Solutions in Healthcare, Texas, USA

**Keywords:** Alzheimer’s disease, African American, Antidepressant usage, Body mass index, Classification and regression tree, Risk factors, Smoking

## Abstract

Alzheimer’s disease (AD) is increasingly prevalent, especially among African American older adults. Despite its widespread nature, accurate and timely diagnosis of AD remains challenging. Addressing the research gap in sociodemographic and cardiovascular risk factor research associated with AD in African American older adults, this study aimed to identify and analyze distinct subgroups within this population that are particularly vulnerable to AD, thereby contributing to the development of targeted interventions and healthcare strategies. This study employs a rigorous methodology utilizing classification and regression tree (CART) analysis to examine data from the 2017 Uniform Data Set (UDS). This approach enables a nuanced analysis of AD susceptibility among African American older adults. The CART analysis revealed significant associations between the studied sociodemographic and cardiovascular risk factors and AD susceptibility among African American older adults. The results indicate the presence of specific subgroups with increased vulnerability to AD, shaped by varying levels of education [relative importance (RI): 100%], antidepressant usage (RI: 83.1%), BMI (RI: 71.2%), use of antipsychotic agents (RI: 35.5%), and age of smoking cessation (RI: 21.5%). These findings underscore the importance of culturally specific research and interventions for addressing AD among African Americans. This study’s findings, revealing significant associations between sociodemographic and cardiovascular risk factors and AD susceptibility among African American older adults, underscore the necessity of developing healthcare policies and interventions specifically tailored to address these risks.

## 1. Introduction

Alzheimer’s disease (AD), a leading form of dementia, is characterized by progressive memory loss and deterioration of conversational skills [1]. The Centers for Disease Control and Prevention (CDC) reported that approximately 5.8 million Americans were affected in 2020, with projections nearing 14 million by 2060, underscoring AD as a growing concern [1]. The disease notably affects 73% of individuals aged 75 and above and is more prevalent among African Americans, affecting 21.3% of those aged 70 or older [2, 3]. This group has a twofold greater incidence of AD than older White individuals. Early-stage AD diagnosis remains a challenge, causing delays in crucial interventions [4, 5, 6]. Research highlights racial disparities in AD diagnosis and progression, with older African Americans bearing more significant burdens than their White counterparts [3, 7, 8]. These disparities stem from systemic inequalities, limited healthcare access, and structural racism in the U.S., necessitating a nuanced understanding of AD within the African American community [5, 7].

We note the following patterns and trends in past studies: 1) Gender and race: The incidence of AD is greater in women older than 79 years [9, 10, 11]. Race, a significant variable in AD incidence, underscores the genetic and socioeconomic factors impacting marginalized populations. Older African Americans are notably more susceptible to AD than non-Hispanic white individuals are [11, 12, 13, 14, 15]. 2) Educational attainment: Higher levels of education correlate with reduced AD risk and symptom severity [16, 17, 18, 19, 20, 21, 22]. However, educational attainment disparities exist between African American and white populations, influencing AD outcomes [23]. 3) Cardiovascular risk factors: Conditions such as hypertension, hypercholesterolemia, smoking, BMI, heart disease status, diabetes status, and stroke history are linked to an increased risk of AD [24, 25, 26, 27, 28, 29, 30, 31, 32, 33, 34, 35, 36, 37, 38, 39, 40]. These factors are particularly relevant for the African American community due to their greater incidence of these conditions. 4) Medication history: The use of antidepressants, anxiolytics, and antipsychotics in AD patients and their varying prevalence across racial lines are areas of ongoing research [41, 42, 43, 44, 45, 46, 47, 48, 49, 50, 51, 52]. Notably, disparities exist in medication use and access to mental health services among different racial groups.

This study aimed to identify vulnerable subgroups within the African American older adult population by examining sociodemographic and cardiovascular risk factors for AD. Using classification and regression tree (CART) analysis, this research aimed to inform culturally tailored prevention and intervention strategies, addressing the unique challenges faced by this community in the context of AD [5, 7].

## 2. Methods

### 2.1. Study sample

This study utilized a cross-sectional design, leveraging comprehensive and longitudinal data from the 2017 Uniform Data Set (UDS) provided by the National Alzheimer’s Coordinating Center (NACC).

The UDS data, collected annually from Alzheimer’s Disease Research Centers (ADRCs), offer rich demographic, clinical, and neurological information, making them an ideal resource for our cross-sectional analysis of AD susceptibility among older African American adults [53]. The UDS data included clinical data from participants across 39 past and present ADRCs in the U.S. Participants were included in this study if they were cognitively normal at baseline and were followed until loss to follow-up or death.

All contributing ADRCs were required to obtain approval from their respective institutional regulatory boards and informed consent from their participants before submitting the data to the NACC.

### 2.2. Classification of AD

A diagnosis of AD was confirmed by a consensus team or physician using the results of a structured clinical history, neuropsychology testing, and validated assessments of symptoms and function. AD-related dementia was diagnosed using the National Institute of Neurological and Communicative Disorders and Stroke and the Alzheimer’s Disease and Related Disorders Association prior to 2015 and the National Institute on Aging and Alzheimer’s Association after 2015. Participants were diagnosed with AD if it was the primary or contributing cause of cognitive impairment, which included participants who had probable or possible dementia of the AD type.

### 2.3. Measures

Table 2 provides an overview of the variables pertinent to AD. The data for our study are from the NACC UDS. Standardized clinical evaluations, which included detailed descriptions previously published, were included in this database. Our study’s dependent variable was AD status, which was captured using the following response options: 0 = ‘no’ and 1 = ‘yes’. The proportion of AD among African American older adults was 31.5%. Gender was treated dichotomously, with response categories coded as 1 = ‘male’ and 2 = ‘female’. In this study, the age variable represents the age at the participants’ first visit, as uniform data on the exact age of AD diagnosis were unavailable. Years of education is a discrete numerical variable measured in total years of completed education. Hypertension, hypercholesterolemia, and congestive heart failure (CHF) were binary categorical variables, denoted by 0 for ‘no’ and 1 for ‘yes’. Age at smoking cessation and the number of smoking years are continuous variables measured in completed years. Body mass index (BMI) was also a continuous variable. Regarding diabetes presence at the initial visit to the Alzheimer’s Disease Center (ADC), the responses were as follows: 0 = ‘absent,’ 1 = ‘recent/active,’ and 2 = ‘remote/inactive.’ However, categories 1 and 2 were amalgamated under the ‘yes’ label for analytical purposes. The stroke history responses were as follows: 0 = ‘absent,’ 1 = ‘recent/active’, and 2 = ‘remote/inactive.’ Categories 1 and 2 were combined for analysis as ‘yes.’ Antidepressant use by subjects was considered, with response options: 0 = ‘did not report use at visit’ and 1 = ‘reported use at visit.’ Similarly, regarding the use of anxiolytics, there were two response categories: 0 = ‘did not report use at visit’ and 1 = ‘reported use at visit.’ Concerning the use of antipsychotic agents, the response categories were as follows: 0 = ‘did not report use at visit’ and 1 = ‘reported use at visit.’ Additionally, we assessed whether subjects experienced sleep apnea during their initial ADC visit, with response categories mirroring diabetes and stroke history. A presumptive etiologic diagnosis of posttraumatic stress disorder (PTSD) was included, with response options of 0 = ‘no’ and 1 = ‘yes.’

### 2.4. Data analysis

#### 2.4.1. Chi-square tests for categorical variables

Initially, we employed chi-square tests to assess the associations between each categorical predictor variable and the binary outcome variable, denoting AD status (0 = No, 1 = Yes). This step was crucial for discerning any significant relationships or dependencies among these variables.

#### 2.4.2. Independent t tests for continuous variables

For continuous variables such as age, age at smoking cessation, and BMI, we utilized independent t tests. This approach enabled us to identify statistically significant differences in these variables between individuals with and without AD.

#### 2.4.3. Multivariate analysis using CART

Subsequently, we performed a more advanced multivariate analysis using the CART algorithm. Our study employed a Minitab 23 for CART analysis, focusing on AD in elderly Asian American individuals. Minitab, known for its robust data analysis capabilities, allowed us to approach the analysis with methodological rigor. We maintained equal prior probabilities for all classes and used the Gini index to split nodes. The minimum misclassification cost determined the optimal tree, which was validated through 10-fold cross-validation. We emphasized transparency and accuracy by providing detailed performance metrics such as log-likelihood, AUC, lift, misclassification cost, and a confusion matrix highlighting key rates for training and test datasets.

## 3. Results

This study included 22,693 African American elderly individuals—5,847 (25.77%) males and 16,846 (74.23%) females. The average years of education was 16.29 ± 3.27, indicating that a group had an average level of education. Other medical and medication histories are included in Table 1.

### 3.1. Bivariate analysis

As shown in Table 2, AD was significantly associated with sociodemographic variables such as age [t(19,887) = −28.47, *p* < 0.001], sex (χ^2^ = 121.45, *p* < 0.001), and years of education [t(22,591) = 20.22, *p* < 0.001]. Other variables also significantly associated with AD included hypercholesterolemia (χ^2^ = 8.63, *p* < 0.003); history of stroke (χ^2^ = 64.28, *p* < 0.001); reported current use of an antidepressant (χ^2^ = 587.21, p < 0.001); reported current use of an anxiolytic, sedative or hypnotic agent (χ^2^ = 4.42, p < 0.036); reported current use of an antipsychotic agent (χ^2^ = 451.29, *p* < 0.001); presumptive etiologic diagnosis of PTSD (χ^2^ = 5.77, *p* < 0.016); age quit smoking [t(5,836) = −4.51, *p* < 0.001); and BMI [t(18,581) = −24.32, *p* < 0.001). However, there were no significant associations between AD and hypertension, congestive heart failure, diabetes, sleep apnea, or the number of years of smoking.

### 3.2. Decision tree results

Our study utilized Minitab 23 for CART analysis, ensuring accuracy and transparency by providing detailed performance metrics. The analysis, conducted with a substantial dataset of 22,693 rows, employed the Gini index for node splitting and a 70/30% training/test set split for model validation. The key findings included an area under the ROC curve (AUC) of 0.6378 in the training cohort and 0.6180 in the testing cohort, with lift values of 1.7947 and 1.6497, respectively. The misclassification costs were 0.7587 for training and 0.7884 for testing, suggesting that our model’s effectiveness was comprehensively evaluated within the CART framework.

**Figure 1.**
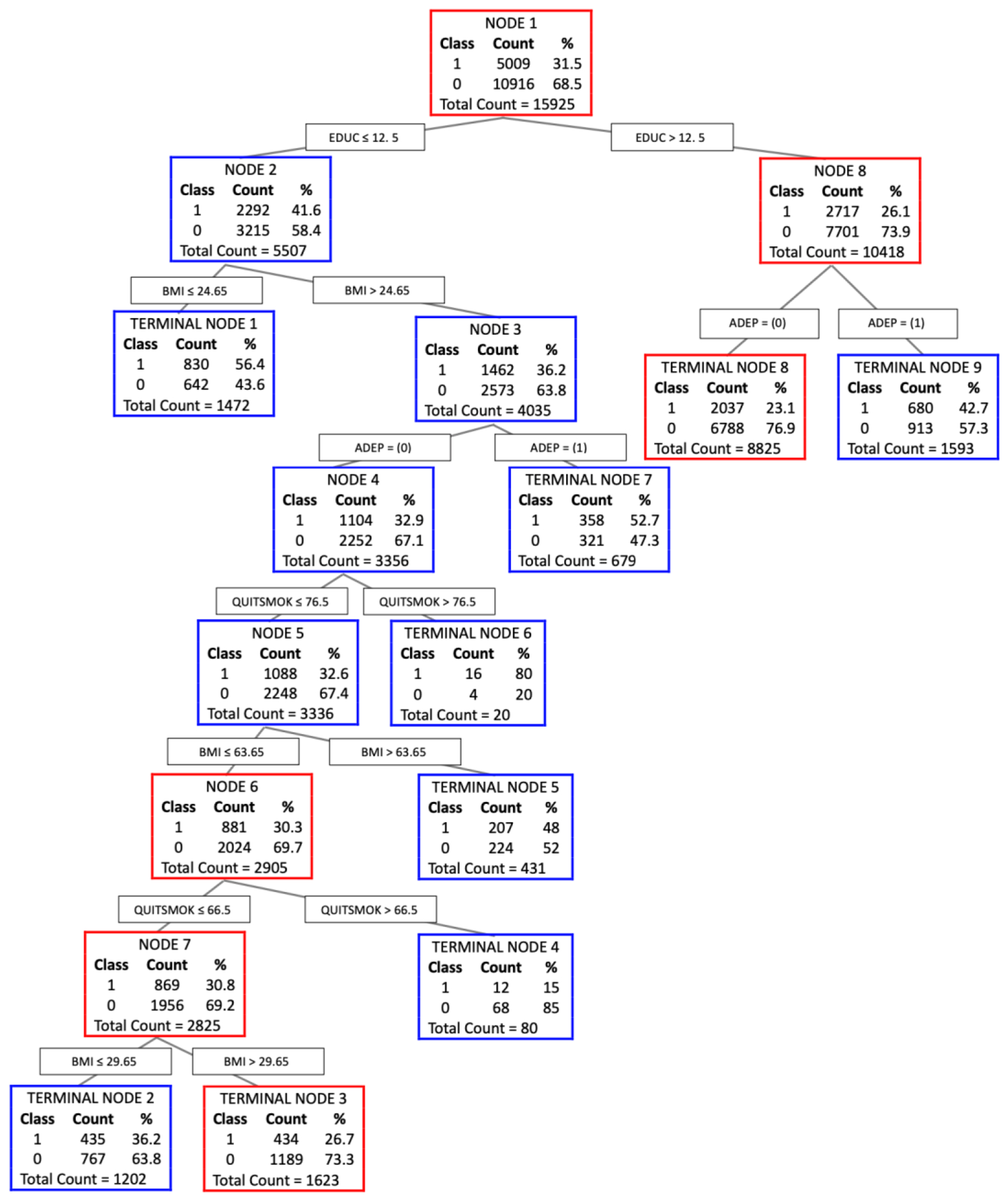
Classification and regression tree (CART) of risk factors for Alzheimer’s disease among African American older adults.

The CART model for identifying factors significantly associated with AD among older African American adults is shown in Figure 1. The CART model creates a tree with a series of branches by splitting the independent variables into two response variables: 1 = ‘yes’ and 0 = ‘no.’ The topmost rectangle in Figure 1 indicates the root node, with 31.5% (n = 5,009) of respondents who reported having AD and 68.5% (n = 10,916) of respondents who did not report having AD. The root node, AD, resulted in eight levels of node splits.

The initial split of all respondents was based on years of education. Of those participants who reported having AD, 26.1% (n = 2,717) reported having more than 12.5 years of education. This node branches to the right based on the use of an antidepressant. Of those participants who reported having an education level greater than 12.5 years, 23.1% reported not having used an antidepressant (n = 2,037), and 42.7% reported using an antidepressant (n = 680). Both branches resulted in terminal nodes. These results suggested that years of education significantly interacts with the use of an antidepressant in relation to AD incidence.

The branch to the left split was based on years of education. Of those who reported AD, 41.6% reported an education level of less than 12.5 years (n = 2,292). This branch was further split by BMI (24.65). Among participants who reported having less than 12.5 years of education, 56.4% reported having a BMI less than 24.65 (n = 830), which resulted in a terminal node, whereas 36.2% of participants reported a BMI greater than 24.65 (n = 1,462), which branched down to the right. These results indicate that years of education significantly interacts with BMI in terms of its relationship with AD incidence.

The second split divides nodes based on the reported use of an antidepressant. Among participants who reported a BMI greater than 24.65, 52.7% of respondents reported using an antidepressant (n = 358), which branched down to the right and resulted in a terminal node. However, 32.9% of the respondents reported not using an antidepressant (n = 1,104), which branched down to the left. These results suggest that years of education is a significant moderator of the relationship between AD and the use of an antidepressant.

The fourth split divides nodes based on the reported age at which the participant quit smoking. Among participants who reported not using an antidepressant, 80% of participants reported that they stopped smoking after the age of 76.5 years (n = 16), which resulted in a terminal node. However, 32.6% of the participants reported that they stopped smoking before the age of 76.5 years (n = 1,088), and these individuals branched down to the left. These results suggest that years of education is a significant moderator of the relationship between AD and the age at which the participant quit smoking.

The fifth split divides nodes based on a BMI of 63.65. Among participants who reported quitting smoking after the age of 76.5, 48% of participants reported having a BMI greater than 63.65 (n = 207), which resulted in a terminal node. However, 30.3% of participants reported having a BMI less than 63.65 (n = 881), which branched down to the left. These results suggest that years of education is a significant moderator of the relationship between AD and BMI.

The sixth split divides nodes based on the reported age at which the participant quit smoking. Among participants who reported having a BMI less than 63.65, 15% of respondents reported that they stopped smoking after the age of 66.5 years (n = 12), which resulted in a terminal node. Additionally, 30.8% of the respondents reported that they stopped smoking before the age of 66.5 years (n = 869), which branched down to the left. These results suggest that years of education is a significant moderator of the relationship between AD incidence and age at which the participant quit smoking.

The seventh split divides nodes based on BMI. Among participants who reported that they quit smoking before the age of 66.5 years, 26.7% of participants reported a BMI greater than 29.65 (n = 434), whereas 36.2% of participants reported a BMI less than 29.65 (n = 435); both branches resulted in terminal nodes.

Figure 1 shows the varied vulnerability profiles for AD within our study population, highlighting how different combinations of predictors influence AD susceptibility levels. The most susceptible group, identified in Terminal Node 6, included individuals with less than 12.5 years of education, a BMI above 24.65, and no past use of antidepressants but who quit smoking after the age of 76.5 years. This group shows an alarmingly high susceptibility to AD, at 80%. The second most vulnerable group, located in Terminal Node 7, was characterized by individuals with less than 12.5 years of education and a BMI less than 24.65 years, exhibiting a notably high incidence of AD and a susceptibility of 56.4%. The third group, in Terminal Node 7, consisted of individuals with less than 12.5 years of education, a BMI above 24.65, and a history of antidepressant use, with 52.7% reporting AD. Last, the fourth group, identified in Terminal Node 5, comprised individuals with less than 12.5 years of education, a BMI over 24.65, no past use of antidepressants, quit smoking before 76.5 years of age, and a BMI above 63.65. Among this group, 48% reported having AD.

The CART software reports “variable importance scores” to determine variable selection. A variable with a score of 100 is indicated as the most influential independent variable for predicting AD, followed by other variables based on their relative importance. Based on these variable importance scores, years of education was the most influential variable (100%), followed by the use of an antidepressant (83.1%), BMI (71.2%), use of antipsychotic medication (33.5%), age of participant quit smoking (21.5%), and sex (3.2%).

## 4. Discussion

The current study aimed to explore the complex relationships between sociodemographic variables, cardiovascular risk factors, and AD among African Americans. This research addresses a significant gap in the literature, which often lacks a comprehensive focus on the African American community. While disparities in AD incidence across racial groups are recognized, many studies have generalized the findings without considering the unique sociocultural, genetic, and environmental factors influencing AD in African Americans [5, 7].

Our study explored how factors such as education, antidepressant use, BMI, and smoking cessation age impact AD risk among older African Americans. We aimed to provide a nuanced understanding of these variables, countering the broad generalizations of previous research [5, 7]. Employing CART analysis, we dissected the intricate interactions of these factors within the African American population, striving to offer insights for tailored interventions and healthcare policies.

Our findings both align with and contradict the literature. Consistently, we observed significant associations between sociodemographic variables such as education and cardiovascular risk factors (hypertension, hypercholesterolemia, heart disease) and AD in African Americans. These findings align with studies identifying education as a protective factor and the established link between cardiovascular health and cognitive function [19, 27, 32, 35, 37]. However, contradictions emerge regarding smoking history and the use of antidepressants, anxiolytics, and antipsychotics. Unlike in some studies, we found no significant association between smoking history and AD [33]. Additionally, our study did not establish a clear link between the use of these medications and AD risk [44, 48, 51]. These discrepancies highlight the complexity of AD risk factors and the need for context-specific research.

Having explored the broader findings and their alignment or contradiction with the literature, we now focus on a unique aspect of our study: vulnerability profiles. The vulnerability profiles depicted in Figure 1 underscore the intricate relationship between demographic and lifestyle factors and susceptibility to AD within our study population. These profiles reveal that distinct combinations of predictors can significantly influence an individual’s risk of developing AD.

One particularly noteworthy finding is the high susceptibility observed in individuals with lower education levels, higher BMI, no past antidepressant use, or late smoking cessation. An alarming 80% of patients in this group exhibited AD susceptibility, highlighting the critical role of these factors in increasing risk.

These vulnerability profiles emphasize the complexity of AD risk and the importance of considering the interplay between various factors [54]. Tailored prevention and intervention strategies should consider these unique profiles to address the specific needs of at-risk individuals, ultimately enhancing our ability to manage and provide care for AD.

### 4.1. Practice implications

The findings from our study on AD risk factors among African Americans have significant implications for clinical practice. These insights are vital for addressing healthcare disparities and promoting equitable healthcare access for this population.

### 4.2. Tailored interventions

The identified associations between education, cardiovascular risk factors, and AD highlight the need for interventions specifically designed for African Americans. Healthcare professionals should focus on educational programs that build cognitive reserves and lifestyle modifications to improve cardiovascular health. These interventions are particularly crucial given the impact of education and cardiovascular health on AD risk [19, 35].

### 4.3. Early detection and screening

Our findings advocate for integrating education level and cardiovascular health assessments into routine screenings for older African American adults. Early identification of high-risk individuals can lead to prompt interventions, potentially slowing AD progression. This strategy aligns with the established roles of education and cardiovascular health in determining AD risk [19, 32].

### 4.4. Implications for vulnerability profiles

The vulnerability profiles identified in our study offer critical insights for personalized healthcare strategies. These profiles underscore the need for healthcare approaches that consider individual risk factors such as education level, BMI, smoking habits, and medication history. Healthcare approaches should be tailored to address the unique combinations of risk factors in different subgroups, as highlighted by vulnerability profiles. In addition, high susceptibility rates in certain groups call for focused screening and preventive measures, allowing earlier and more effective intervention.

### 4.5. Study limitations and future study suggestions

While this study provides valuable insights into the association between sociodemographic variables, cardiovascular risk factors, and AD among African Americans, several limitations warrant consideration. These limitations offer directions for future research to build upon and enhance our understanding of this complex relationship. First, the cross-sectional design of this study restricts our ability to establish causal relationships between variables. Longitudinal studies could be beneficial for capturing the temporal dynamics of the identified associations and providing a clearer picture of how sociodemographic factors and cardiovascular risk factors contribute to AD susceptibility over time.

Second, the reliance on self-reported data introduces potential biases such as recall bias and social desirability bias. Incorporating objective measures and biomarkers could offer a more accurate assessment of cardiovascular risk factors and cognitive status, thereby strengthening the validity of the findings.

Another limitation pertains to the lack of consideration of genetic factors. While this study focused on sociodemographic and cardiovascular risk factors, genetics plays a significant role in AD susceptibility. Future research could integrate genetic analyses to comprehensively understand the interplay between genetics, sociodemographic variables, and cardiovascular risk factors in shaping AD risk [20].

Furthermore, the study predominantly focused on older African American adults, limiting the generalizability of the findings to other age groups and racial populations. Exploring the associations across different age cohorts and racial backgrounds could offer insights into potential variations in AD risk factors and contribute to a more comprehensive understanding of the broader population. Investigating the mediating mechanisms underlying the identified associations could provide valuable insights for future research. For example, exploring the role of inflammation, oxidative stress, and vascular health as mediators of the relationship between cardiovascular risk factors and AD risk among African Americans could enhance our understanding of the underlying pathways . [40] Additionally, qualitative research approaches could supplement quantitative findings by delving into the contextual factors influencing AD risk among African Americans. Qualitative studies could explore the lived experiences, cultural beliefs, and social determinants contributing to the observed associations, providing a more comprehensive perspective [55].

## 5. Conclusions

This study significantly advances the understanding of AD susceptibility among African American older adults. Through detailed sociodemographic and cardiovascular risk factor analysis via CART analysis, we identified distinct subgroups within this population that exhibited increased vulnerability to AD. These findings emphasize the critical need for personalized healthcare approaches and interventions tailored to these specific risk profiles. Our research highlights the importance of targeted strategies for mitigating AD risk. This finding underscores the value of culturally sensitive healthcare practices in effectively addressing the unique challenges faced by African Americans in AD.

## Figures and Tables

**Table 1.** Study participant demographics (n = 22,693)

Demographics	N (%)
Sex, men	5,847.00 (25.77%)
Sex, female	16,846.00 (74.23%)
Age (in years), Mean ± SD	76.83 ± 7.27
Years of education (in years), Mean ± SD	14.36 ± 3.24
*Cardiovascular risk factors*	
Hypertension	6,549.00 (28.86%)
Hypercholesterolemia	5,191.00 (22.87%)
Age of quit smoking, Mean ± SD	46.60 ± 14.66
Total years smoked cigarettes, Mean ± SD	12.15 ± 17.31
BMI, Mean ± SD	29.43 ± 6.28
Congestive heart failure	384.00 (1.69%)
Diabetes	4,444.00 (19.58%)
History of stroke	1,154.00 (5.09%)
PTSD	94.00 (0.41%)
Apnea	368.00 (1.62%)
*Medication history*	
Antidepressant use	3,605.00 (15.89%)
Anxiolytic use	1,752.00 (7.72%)
Antipsychotic use	681.00 (3.00%)
Incident AD	7100.00 (31.29%)

**Table 2.** Differential characteristics of risk factors according to AD status (n = 22,693)

Variables		AD Status		*chi-square/t value*	*p* value **
		No, AD	Yes, AD		

		N (%)	N (%)		
Sex	Male	36,810 (76.4%)	2,166 (30.51%)	121.45	< 0.001 **

	Female	11,912 (23.6%)	4,934 (69.49%)		

Hypertension	No	1,831 (27.40%)	617 (26.66%)	0.47	0.494

	Yes	4,852 (72.60%)	1,697 (73.34%)		

Hypercholesterolemia	No	2,829 (42.68%)	895 (39.15%)	8.63	0.003 **

	Yes	3,800 (57.32%)	1,391 (60.85%)		

Congestive heart failure	No	6,405 (95.96%)	2,193 (95.06%)	3.37	0.067

	Yes	270 (4.04%)	114 (4.94%)		

Diabetes	No	7,320 (71.19%)	3,854 (72.24%)	1.92	0.166

	Yes	2,963 (28.81%)	1,481 (27.76%)		

History of stroke	No	7,812 (92.45%)	3,846 (88.17%)	64.28	< 0.001 **

	Yes	638 (7.55%)	516 (11.83%)		

Use of antidepressant	No	13,511 (87.90%)	5,248 (75.05%)	587.21	< 0.001 **

	Yes	1,860 (12.10%)	1,745 (24.95%)		

Use of anxiolytic	No	14,206 (92.42%)	6,406 (91.61%)	4.42	0.036 **

	Yes	1,165 (7.58%)	587 (8.39%)		

Use of antipsychotic agent	No	15,156 (98.60%)	6,527 (93.34%)	451.29	< 0.001 **

	Yes	215 (1.40%)	466 (6.66%)		

Apnea	No	1,127 (80.79%)	454 (81.95%)	0.35	0.555

	Yes	268 (19.21%)	100 (18.05%)		

PTSD	No	6,632 (98.81%)	2,302 (99.40%)	5.77	0.016 **

	Yes	80 (1.19%)	14 (0.60%)		

Age (in years), Mean ± SD		75.83 ± 6.99	78.89 ± 7.41	−28.47	< 0.001 **

Years of education (in years), Mean ± SD		14.65 ± 3.07	13.72 ± 3.49	20.22	< 0.001 **

Age of quitting smoking (in years), Mean ± SD		45.96 ± 14.49	47.79 ± 14.91	−4.51	< 0.001 **

Total years of smoking (in years), Mean ± SD		12.20 ± 17.14	12.06 ± 17.65	0.46	0.324

BMI, Mean ± SD		30.15 ± 6.34	27.74 ± 5.80	−28.47	< 0.001 **

* Discrepancies in total counts per variable are due to non-responses, reflecting actual data received.

** Significant value (*p* < 0.05).

## Data Availability

The research was conducted using data from the 2017 Uniform Data Set (UDS), provided by the National Alzheimer’s Coordinating Center (NACC). This comprehensive dataset is publicly available and can be accessed through the NACC website at naccdata.org.
